# *Vibrio vulnificus* VvhA induces NF-*κ*B-dependent mitochondrial cell death via lipid raft-mediated ROS production in intestinal epithelial cells

**DOI:** 10.1038/cddis.2015.19

**Published:** 2015-02-19

**Authors:** S-J Lee, Y H Jung, S Y Oh, E J Song, S H Choi, H J Han

**Affiliations:** 1 Department of Veterinary Physiology, College of Veterinary Medicine, Research Institute for Veterinary Science, and BK21 PLUS Creative Veterinary Research Center, Seoul National University, Seoul 151-741, South Korea; 2 National Research Laboratory of Molecular Microbiology and Toxicology, Department of Agricultural Biotechnology, and Center for Food Safety and Toxicology, Seoul National University, Seoul 151-921, South Korea

## Abstract

The Gram-negative bacterium *Vibrio vulnificus* produces hemolysin (VvhA), which induces cytotoxicity in mammalian cells. However, our understanding of the cytotoxic mechanism and the modes of action of VvhA are still fragmentary and incomplete. The recombinant protein (r) VvhA (50 pg/ml) significantly induces necrotic cell death and apoptosis in human intestinal epithelial (INT-407) cells. The apoptotic cell death induced by rVvhA is highly susceptible to the sequestration of cholesterol by methyl-*β*-cyclodextrin, whereas for necrotic cell death, this shows a marginal effect. We found that rVvhA induces the aggregation of lipid raft components coupled with NADPH oxidase enzymes, in which rVvhA increased the interaction of NADPH oxidase 2 (NOX2, gp91^phox^) with a cytosolic protein NCF1 (p47^phox^) to facilitate the production of reactive oxygen species (ROS). rVvhA uniquely stimulated a conventional PKC isoform PKC*α* and induced the phosphorylation of both ERK and JNK, which are responsible for the activation of transcription factor NF-*κ*B. rVvhA induced an NF-*κ*B-dependent imbalance of the Bcl-2/Bax ratio, the release of mitochondrial cytochrome *c*, and caspase-3/-9 activation during its promotion of apoptotic cell death. In addition, rVvhA has the ability to inhibit the expression of cell cycle-related proteins, such as CDK2, CDK4, cyclin D1, and cyclin E. These results demonstrate that rVvhA induces NF-*κ*B-dependent mitochondrial cell death via lipid raft-mediated ROS production by the distinct activation of PKC*α* and ERK/JNK in intestinal epithelial cells.

Intestinal epithelial cell death is a host defense response that eliminates damaged cells as well as pathogens to maintain gut homeostasis.^1^ However, many bacterial pathogens eventually elicit epithelial cells death and disrupt the gut barrier function to propagate persistent bacterial colonization.^2, 3, 4, 5^ *Vibrio vulnificus* is a food-borne pathogenic bacterium that causes septicemia, necrotizing wound infections, or gastroenteritis.^6^ Many secreted and cell-associated virulence factors of *V. vulnificus* have been shown to induce fulminating and destructive actions in animal tissues.^7^ Among the secreted virulence factors of *V. vulnificus*, cytolytic pore-forming hemolysin (VvhA)^8^ and multifunctional autoprocessing RTX (MARTXVv)^9^ have been shown to promote intestinal colonization, which can be responsible for the death of the host during bacterial infection. Despite the functional role of the MARTXVv, which has been thoroughly studied owing to its significant contribution to mouse lethality studies, the mechanism of the cytotoxicity of VvhA remains a topic of much debate. VvhA is a 51-kDa water-soluble pore-forming toxin that has been shown to induce cytotoxicity through oligomerization at cholesterol-enriched membrane domains known as lipid rafts.^10, 11^ However, it has been reported that lipid rafts may not be involved in the cytotoxicity of VvhA.^12^ Thus the cytotoxic mechanism of VvhA and how it acts with regard to lipid rafts remain controversial and vaguely understood issues.

Apoptosis is a cell death mechanism accompanied by a highly complex cellular events mediated by the caspase cascade that results in chromatin condensation, DNA fragmentation, cytoplasmic membrane blebbing and cell shrinkage.^13^ In contrast, necrosis is associated with caspase-independent inflammation characterized by membrane rupture, nuclear swelling, and the release of cellular contents.^14^ Bacterial pathogens can induce apoptosis and/or necrosis by a variety of direct and indirect mechanisms and eventually disturb a fine balance between apoptosis and necrosis that may be a key element in the development of some diseases.^15^ Given that VvhA has the ability to induce two general modes of cell death, apoptosis^16^ and necrosis,^17^ it is important to know how VvhA selectively regulates apoptosis and necrosis in determining the mode of host cell death.

Many enteric bacterial pathogens such as *Salmonella typhimurium*,^2, 3^ *Helicobacter pylori*,^4^ and *Enteropathogenic Escherichia coli* (EPEC)^5^ are known to induce apoptosis through unique cellular mechanisms that regulate intrinsic/extrinsic environmental factors, such as oxidative stress, the mitogen-activated protein kinase (MAPK) signaling pathway, mitochondrial damage, and caspase-3 activation. Membrane lipid rafts are another important element in the initiation of many apoptotic signaling pathways, having a main role in the interaction between bacterial pathogens and hosts.^18, 19^ Emerging evidence has shown that lipid rafts form unique functional redox signaling platforms that are responsible for the production of reactive oxygen species (ROS) via the clustering of the NADPH oxidase (NOX) family in promoting apoptotic cell death.^20, 21, 22^ Although VvhA is also known to induce apoptosis via ROS production in several cells, our understanding of the apoptotic mechanism and the modes of action of VvhA during intestinal infection remains fragmentary and incomplete. In this study, therefore, we investigate both the role of VvhA in promoting the cell death of intestinal epithelial cells and related signaling pathways.

## Results

### VvhA induces apoptotic cell death as well as necrosis

**Figure 1:**
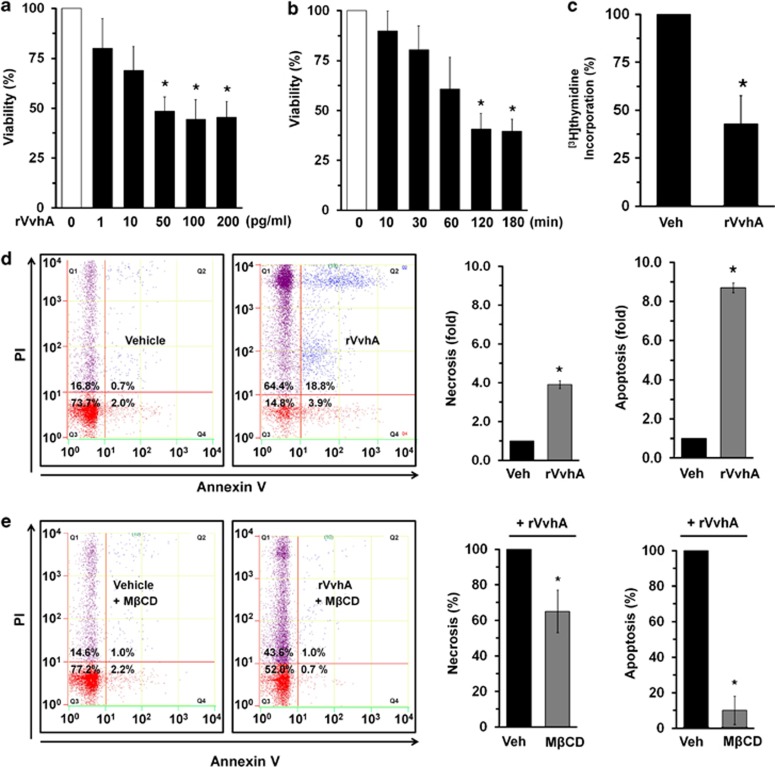
VvhA induces necrotic cell death as well as apoptosis. (**a**) Dose responses of rVvhA for 120 min in MTT assay are shown. Data represent means±S.E. *n*=5. *P<0.05 *versus* 0 pg/ml. (**b**) Time responses of 50 pg/ml of rVvhA in MTT assay are shown. Error bars represent the means±S.E. *n*=5. *P<0.01 *versus* 0 min. (**c**) INT-407 cells were synchronized by serum starvation for 24 h and treated with 50 pg/ml rVvhA for 120 min. [^3^H]thymidine incorporation was determined. Data represents the means±S.E. of four independent experiments for each condition. *P<0.05 *versus* Veh (boiled rVvhA, 200pg/ml). (**d**) INT-407 cells were incubated with 50 pg/ml of rVvhA for 120 min. Percentages of necrosis, survival, and apoptosis were measured by using PI/Annexin V staining and flow cytometry (left panel). PI^+^/Annexin V^−^ cells (Q1) were considered necrotic, PI^+^/Annexin V^+^ double-positive cells (Q2) were considered late apoptotic, PI^−^/Annexin V^−^ cells (Q3) were considered alive, and PI^−^/Annexin V^+^ cells (Q4) were considered early apoptotic. Quantitative analysis of the fold changes of necrotic (Q1) and apoptotic (Q2+Q4) cells by fluorescence-activated cell sorting (FACS) analysis is shown (right panel). Error bars represent the means±S.E. (*n*=5). *P<0.05 *versus* Veh (vehicle, boiled rVvhA, 200pg/ml). (**e**) INT-407 cells were pretreated with M*β*CD (0.1 mM) for 60 min prior to rVvhA exposure for 120 min. Percentages of necrosis, survival, and apoptosis were measured by using the PI/Annexin V staining and flow cytometry (left panel). Quantitative analysis of the percentage of necrotic (Q1) and apoptotic (Q2+Q4) cells by FACS analysis is shown (right panel). Error bars represent the means±S.E. (*n=5*). *P<0.05 *versus* Veh (vehicle, PBS)+rVvhA

To find the cytotoxic mechanism of VvhA, human intestinal epithelial (INT-407) cells were exposed to various concentrations (0–200 pg/ml) of rVvhA for 2 h. rVvhA significantly induced cytotoxicity of INT-407 cells from 50 to 200 pg/ml, compared with the cells with no treatment (Figure 1a). An increase in cytotoxicity was observed after 2 h of incubation with 50 pg/ml of rVvhA (Figure 1b). In addition, 50 pg/ml rVvhA was able to induce cytotoxicity for most population of cells (~90%) at 24 h (Supplementary Figure S1). The results after the [^3^H]thymidine incorporation of INT-407 cells also revealed that 50 pg/ml of rVvhA significantly attenuated the level of DNA synthesis, compared with the vehicle (Figure 1c). In addition, flow cytometric analysis showed that rVvhA significantly induced the necrotic cell death (a 3.9±0.2-fold increase compared with the vehicle) as well as apoptosis (a 8.7±0.4-fold increase compared with the vehicle) of INT-407 cells (Figure 1d), suggesting that rVvhA might have distinct pathways to induce cell death. We further confirmed the apoptosis/necrosis-promoting effect of rVvhA by using another reagent that monitors the apoptotic cells with phosphatidylserine marker as well as the necrotic cells with 7-aminoactinomycin D (7-AAD), which has a strong affinity for GC-rich regions of DNA. As shown in Supplementary Figure S2, we found that rVvhA is able to induce apoptosis as well as necrosis. Consistent with the results of flow cytometric analysis, rVvhA was essential for triggering the apoptotic cell death rather than the necrosis. This result suggests that the functional role of rVvhA to induce cell death is reproducible in different assays. Cholesterol has been thought to be one of the cellular receptors of VvhA.^11^ To confirm the structural importance of membrane lipid rafts in the rVvhA-mediated signaling pathway, we employed the lipid raft sequester methyl-*β*-cyclodextrin (M*β*CD), which is known to deplete cholesterol from the cell membrane. Interestingly, M*β*CD has relatively more inhibitory potency on the apoptotic cell death (a 91±8% decrease compared with the rVvhA alone) than the necrotic cell death (a 34±9% decrease compared with the rVvhA alone) (Figure 1e), suggesting that rVvhA in acting through lipid raft is essential for triggering the apoptotic cell death rather than the necrosis.

### Involvement of a lipid raft and NOX2-mediated ROS production in apoptotic cell death

**Figure 2:**
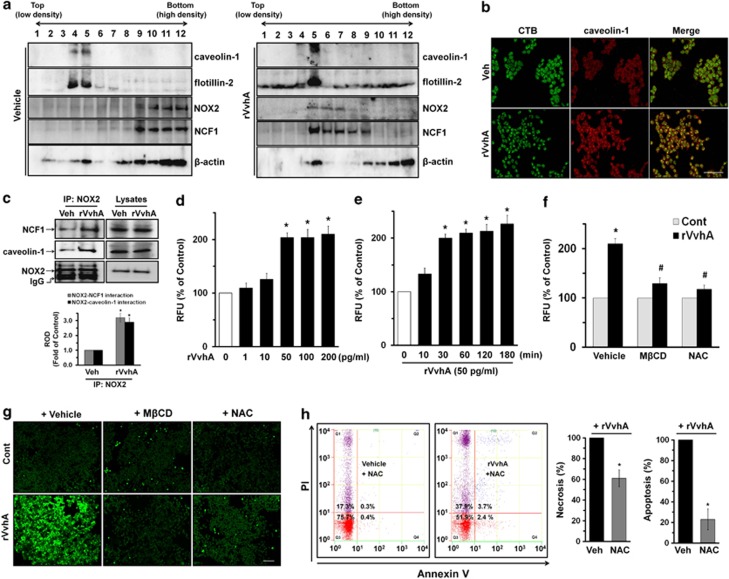
Involvement of a lipid raft and NOX2-mediated ROS production in apoptotic cell death. (**a**) INT-407 cells were incubated in the presence of rVvhA (50pg/ml) for 30 min. Caveolin-enriched membrane fractions were prepared by discontinuous sucrose density gradient fractionation, and the location of caveolin-1, flotillin-2, NOX2, and NCF1 was determined by western blotting analysis. *n*=3. Vehicle (boiled rVvhA, 200pg/ml) (**b**) The increased co-localization of CTB (green) with caveolin-1 (red) was determined by confocal microscopy using immunofluorescence staining. Scale bars, 100 *μ*m (magnification, × 400). *n*=3. Veh (boiled rVvhA, 50pg/ml) (**c**) The cells were incubated in the presence of rVvhA (50 pg/ml) for 30 min and then harvested. NOX2 was immunoprecipitated with an anti-NOX2 antibody, and co-immunoprecipitated NCF1 and caveolin-1 were detected by using anti-NCF1 and anti- caveolin-1 antibodies (left side). Expression of NOX2, NCF1, and caveolin-1 in total cell lysates is shown in the right side. Error bars represent the mean±S.E. *n*=3. *P<0.05 *versus* Veh (boiled rVvhA, 200pg/ml). (**d**) Dose responses of rVvhA for 30 min in ROS production are shown. Data represent means±S.E. of five independent experiments with triplicate dishes. *P<0.01 *versus* 0 pg/ml. (**e**) Time responses of 50 pg/ml of rVvhA in ROS production are shown. Error bars represent the mean±S.E. *n*=5. *P<0.01 *versus* 0 min. (**f**) Cells were pretreated with either M*β*CD for 60 min or *N*-acetylcysteine (NAC, 10 *μ*M) for 30 min prior to rVvhA exposure for 30 min. The level of ROS production is shown. Error bars represent the means±S.E. (*n*=5). *P<0.05 *versus* Cont (boiled rVvhA, 200 pg/ml). ^#^*P*<0.05 *versus* vehicle (PBS)+rVvhA. (**g**) Cells were pretreated with M*β*CD and NAC prior to rVvhA exposure for 30 min and treated with 10 mM DCF-DA. ROS production (green) was determined by confocal microscopy. Scale bars, 50 *μ*m (magnification, × 200). *n*=3. (**h**) INT-407 cells were pretreated with NAC for 30 min prior to rVvhA exposure for 120 min. Percentages of necrosis, survival, and apoptosis were measured by using PI/Annexin V staining and flow cytometry (top panels). Quantitative analysis of the percentage of necrotic (Q1) and apoptotic (Q2+Q4) cells by fluorescence-activated cell sorting analysis is shown (bottom panels). Error bars represent the means±S.E. (*n*=5). *P<0.05 *versus* Veh (PBS)+rVvhA. RFU, relative fluorescence units; ROD, relative optical density

To know how VvhA mediates apoptosis via lipid raft signaling, we determined the effect of rVvhA on the membrane location of caveolin-1 and flotillin-2, which are the major markers of lipid rafts, by means of discontinuous sucrose density-gradient centrifugation. Figure 2a shows that caveolin-1 and flotillin-2 were detected in fractions 4 and 5. Interestingly, the cells treated with rVvhA induced recruitment of caveolin-1 and flotillin-2 into fraction 5, suggesting that rVvhA regulates cellular location of caveolin-1 and flotillin-2. Moreover, the subunits of NADPH oxidases (NOX) enzymes, NOX2 (gp91^phox^) and NCF1 (p47 ^phox^), were highly enriched in the fractions 9–12. However, rVvhA treatment resulted in translocations of NOX2 and NCF1 into fractions 5–8, including lipid rafts. The effect of rVvhA on the membrane location of caveolin-1 was further visualized by staining the caveolin-1 and lipid raft marker molecule, cholera toxin subunit B (CTB). As shown in Figure 2b, rVvhA significantly increased the co-localization of CTB with caveolin-1. In addition, we found that NOX2 co-immunoprecipitated with NCF1 as well as caveolin-1, and importantly, that the interaction with NCF1 and caveolin-1 was enhanced by the rVvhA treatment, suggesting that rVvhA induces clustering of lipid raft molecules and NOX enzymes (Figure 2c). In addition, a significant increase in the ROS level appeared after incubation with 50–200 pg/ml for 30 min compared with the vehicle alone (Figure 2d). The increase in ROS production was augmented between 30 and 180 min after incubation with 50 pg/ml of rVvhA (Figure 2e). To clarify the involvement of lipid rafts in rVvhA-mediated ROS production, cells were pretreated with a M*β*CD or an antioxidant, *N*-acetylcysteine (NAC). As shown in Figure 2f, the production of ROS by rVvhA was significantly blocked by the treatment with M*β*CD as well as NAC. Increased levels of ROS after treatment with 50 pg/ml of rVvhA were visualized by staining INT-407 cells with a fluorescent dye, 2',7'-dichlorofluorescein diacetate (DCF-DA) (Figure 2g). However, a pretreatment with M*β*CD or NAC significantly blocked the ROS production induced by rVvhA. In addition, NAC has relatively more inhibitory potency on the apoptotic cell death (a 78±11% decrease compared with the rVvhA alone) than the necrotic cell death (a 39±8% decrease compared with the rVvhA alone) (Figure 2h).

### Essential role of protein kinase C (PKC) in apoptosis

**Figure 3:**
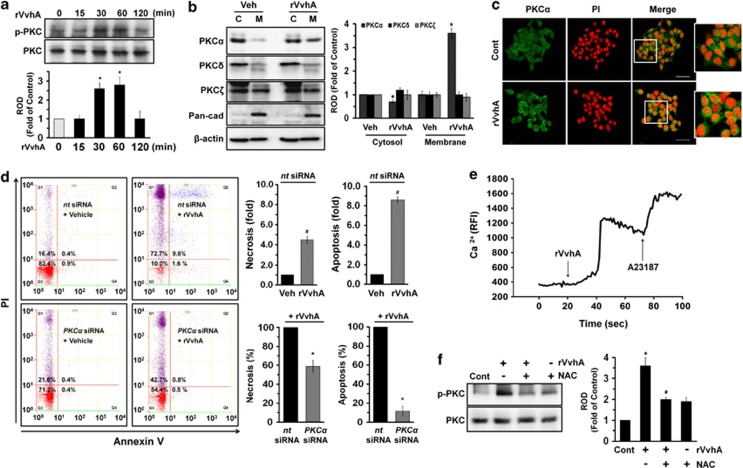
Essential role of PKC in apoptosis. (**a**) Phosphorylation of PKC in cells treated with rVvhA is shown. Error bars represent the mean±S.E. (*n*=4). *P<0.05 *versus* 0 min. (**b**) Membrane translocation of PKC isoforms in cells treated with rVvhA for 30 min was determined by western blotting analysis. The Pan-cad (cadherin) was used as a plasma membrane control. Error bars represent the means±S.E. from three independent experiments involving triplicates. *P<0.05 *versus* Veh (boiled rVvhA, 200pg/ml). C, cytosol; M, membrane. (**c**) Membrane translocation of PKC*α* (green) was determined by confocal microscopy using immunofluorescence staining. PI was used for nuclear counterstaining (red). Scale bars, 100 *μ*m (magnification, × 400). *n*=3. Cont (boiled rVvhA, 200pg/ml). (**d**) INT-407 cells transfected with siRNAs for non-targeting (*nt*) control and *PKCα* were incubated with rVvhA (50 pg/ml) for 120 min. Percentages of necrosis, survival, and apoptosis were measured by using PI/Annexin V staining and flow cytometry (left panels). Quantitative analysis of the percentage of necrotic (Q1) and apoptotic (Q2+Q4) cells by fluorescence-activated cell sorting analysis is shown (right panels). Error bars represent the means±S.E. (*n*=4). ^#^*P*<0.05 *versus* *nt* siRNA+Veh (boiled rVvhA, 200pg/ml). *P<0.05 *versus nt* siRNA+rVvhA. (**e**) The cells were loaded with 2 *μ*M Fluo-3/AM in serum-free medium for 40 min and treated with rVvhA (50 pg/ml). Cells were then treated A23187 (10 *μ*M, Ca^2+^ionophore) as a positive control. Changes in [Ca^2+^]i were monitored by confocal microscopy, and data are expressed as relative fluorescence intensity (RFI, *F*/*F*0%, arbitrary unit). *n*=3. (**f**) Cells were pretreated with NAC (10 *μ*M) prior to rVvhA exposure for 30 min. Phosphorylation of PKC is shown. Error bars represent the means±S.E. (*n*=5). *P<0.05 *versus* Cont (boiled rVvhA, 200 pg/ml). ^#^*P*<0.05 *versus* rVvhA alone. ROD, relative optical density

ROS have an important role as signal messengers in regulating cellular functions through the activation of PKC.^23^ We found that rVvhA significantly induces PKC phosphorylation between 30 and 60 min (Figure 3a). In addition, Figure 3b showed that rVvhA stimulated membrane (M) translocation of PKC*α* from cytosol (C) compartment in the INT-407 cells treated with 50 pg/ml of rVvhA for 30 min, whereas it did not have any effect on the other PKC isoforms such as PKC*δ* and PKC*ζ*. The membrane translocation of PKC*α* was further confirmed by immunofluorescence staining in rVvhA-treated INT-407 cells (Figure 3c). In addition, knockdown of *PKCα* by small interfering RNA (siRNA) showed inhibitory effect on cytotoxicity causing apoptosis (a 88±2% decrease compared with the rVvhA alone) rather than necrosis (a 39±6% decrease compared with the rVvhA alone) induced by rVvhA (Figure 3d). We also assessed the involvement of calcium influx during the apoptosis induced by rVvhA. As shown in Figure 3e, 50 pg/ml of rVvhA induced an increase in calcium influx [Ca^2+^]i. A Ca^2+^ ionophore (A23187), which increases [Ca^2+^]i, was used as a positive control to validate the results. Interestingly, the silencing of ROS with NAC significantly blocked the rVvhA-induced phosphorylation of PKC (Figure 3f). These data suggest a functional role of PKC*α* in regulating rVvhA-mediated apoptosis.

### Regulatory effect of VvhA on MAPK activation and nuclear factor-kappa B (NF-*κ*B) phosphorylation

**Figure 4:**
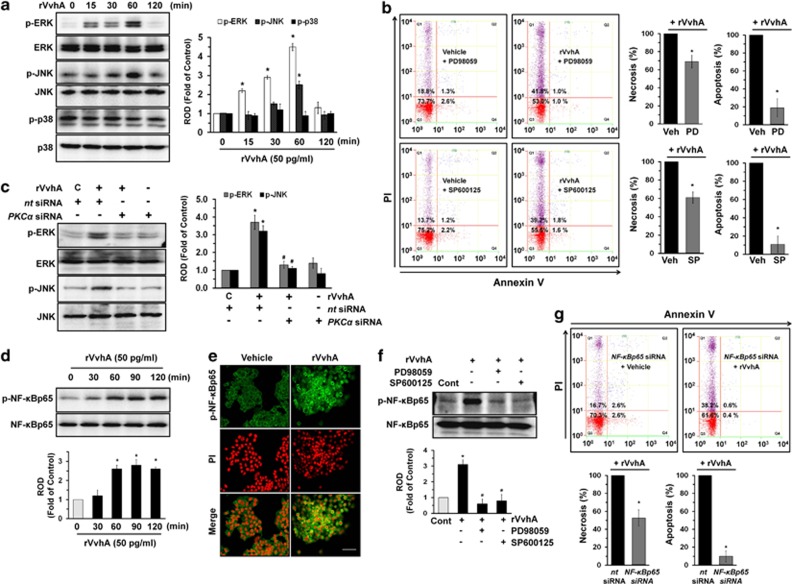
Regulatory effect of VvhA on MAPK activation and NF-*κ*B phosphorylation. (**a**) INT-407 cells were incubated in the presence of rVvhA (50 pg/ml) for various times (0–120 min) and then harvested. Total protein was extracted and blotted with p-ERK, p-JNK, p-p38, ERK, JNK, and p38 antibodies. Error bars represent the means±S.E. from four independent experiments involving triplicates. *P<0.01 *versus* 0 min. (**b**) Cells were pretreated with ERK inhibitor PD98059 (10 *μ*M) and JNK inhibitor SP600125 (10 *μ*M) for 30 min prior to rVvhA (50 pg/ml) exposure for 60 min. Percentages of necrosis, survival, and apoptosis were measured by using PI/Annexin V staining and flow cytometry (left panels). Quantitative analysis of the percentage of necrotic (Q1) and apoptotic (Q2+Q4) cells by fluorescence-activated cell sorting (FACS) analysis is shown (right panels). Error bars represent the means±S.E. (*n*=4). *P<0.05 *versus* Veh (PBS)+rVvhA. (**c**) INT-407 cells transfected with siRNAs for non-targeting (*nt*) control and *PKCα* were incubated with rVvhA (50 pg/ml) for 60 min. Error bars represent the mean±S.E. (*n* =3). *P<0.01 *versus nt* siRNA+C (boiled rVvhA, 200 pg/ml). ^#^*P*<0.01 *versus nt* siRNA+rVvhA. (**d**) The cells were incubated in the presence of rVvhA (50 pg/ml) for various times (0–120 min) and then the phosphorylation of NF-*κ*Bp65 was determined by western blotting with p-NF-*κ*Bp65 and NF-*κ*Bp65 antibodies. Error bars represent the mean±S.E. (*n*=3). *P<0.01 *versus* 0 min. (**e**) The cells were treated with rVvhA (50 pg/ml) for 60 min. p-NF-*κ*Bp65 (green) was detected by immunostaining with p-NF-*κ*Bp65 antibody. The increased nuclear expression of p-NF-*κ*Bp65 was observed. Scale bars represent 50 *μ*m (magnification, × 400). PI was used for nuclear counterstaining (red). *n*=3. Vehicle (boiled rVvhA, 200pg/ml). (**f**) Cells were pretreated with ERK inhibitor PD98059 (10 *μ*M) and JNK inhibitor SP600125 (10 *μ*M) for 30 min prior to rVvhA (50 pg/ml) exposure for 60 min. The phosphorylation of NF-*κ*Bp65 was determined by western blotting with p-NF-*κ*Bp65 and NF-*κ*Bp65 antibodies. Error bars represent the mean±S.E. (*n* =4). *P<0.05 *versus* Cont (boiled rVvhA, 200 pg/ml). ^#^*P*<0.05 *versus* rVvhA alone. (**g**) INT-407 cells transfected with siRNAs for non-targeting (*nt*) control and *NF-κBp65* were incubated with rVvhA (50 pg/ml) for 60 min. Percentages of necrosis, survival, and apoptosis were measured by using PI/Annexin V staining and flow cytometry (top panels). Quantitative analysis of the percentage of necrotic (Q1) and apoptotic (Q2+Q4) cells by FACS analysis is shown (bottom panels). Error bars represent the means±S.E. (*n*=4). *P<0.05 *versus nt* siRNA+rVvhA. PD, PD98059; ROD, relative optical density; SP, SP600125

We then determined how rVvhA links to the activation of MAPKs, which are interesting candidates as downstream mediators of ROS and PKC in the regulation of apoptotic cell death. rVvhA increased the phosphorylation of extracellular signal-regulated kinases (ERKs) between 15 and 60 min or c-Jun N-terminal kinase (JNK) at 60 min (Figure 4a) but did not affect the phosphorylation of p38 MAPK. A pretreatment with the ERK inhibitor PD98059 or JNK inhibitor SP600125 significantly blocked the cytotoxic effect of rVvhA, where PD98059 and SP600125 have more inhibitory potency on apoptotic cell death than a necrosis (Figure 4b). To provide more evidence of the involvement of ERK and JNK, we further studied whether knockdown of ERK1/2 and JNK regulates apoptotic/necrotic cell death in rVvhA-treated INT-407 cells (Supplementary Figure S3). Similar to the inhibitory effect of PD98059 and SP600125 on cytotoxic effect of rVvhA, silencing of *ERK1/2* and *JNK* by siRNAs showed significant inhibitory effect on the apoptotic cell death rather than the necrotic cell death. In addition, the phosphorylation of ERK and JNK evoked by a treatment with rVvhA was markedly inhibited by knockdown of *PKCα* by siRNA (Figure 4c). These data represent an evidence that the phosphorylation of ERK and JNK is regulated by the activation of PKC, as required for induction of apoptosis. We further examined the role of rVvhA in activation of NF-*κ*B, which is a direct transcriptional target for apoptotic signaling pathway. As shown in Figure 4d, NF-*κ*B phosphorylation increased between 60 and 120 min after incubation with 50 pg/ml of rVvhA.

The increased accumulation of NF-*κ*B phosphorylation in the nucleus was further confirmed by immunofluorescence staining and counter-labeling with propidium iodide (PI) (Figure 4e). Pretreatment with the ERK inhibitor PD98059 and JNK inhibitor SP600125 significantly blocked rVvhA-induced phosphorylation of NF-*κ*B (Figure 4f). In addition, knockdown of *NF-κBp65* by siRNA also showed significant inhibitory effect on the apoptotic cell death rather than the necrotic cell death (Figure 4g).

### Regulatory effect of VvhA on mitochondria-mediated apoptotic cell death

**Figure 5:**
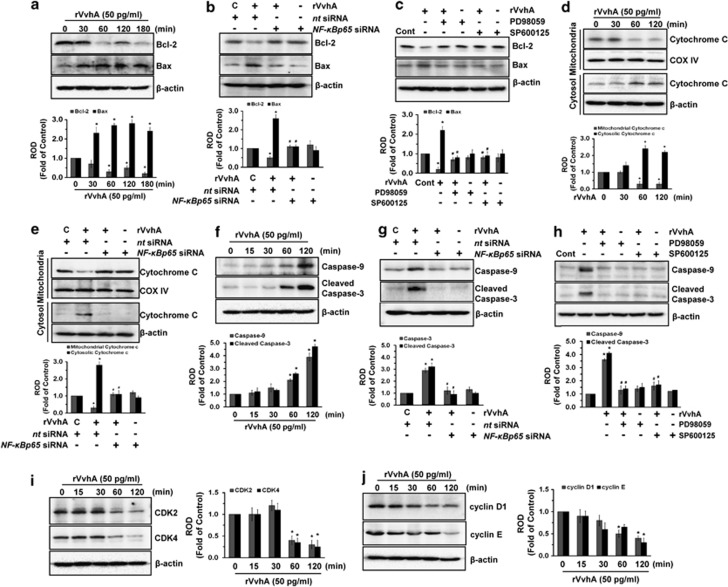
Regulatory effect of VvhA on mitochondria-mediated apoptotic cell death. (**a**) INT-407 cells were incubated in the presence of rVvhA (50 pg/ml) for various times (0–180 min) and then harvested. Total protein was extracted and blotted with Bcl-2 and Bax antibodies. Error bars represent the means±S.E. (*n*=4). *P<0.05 *versus* 0 min. INT-407 cells were (**b**) transfected with siRNA for *NF-κBp65* or (**c**) pretreated with inhibitors of ERK (PD98059,10 *μ*M) and JNK (SP600125, 10 *μ*M) prior to rVvhA (50 pg/ml) exposure for 120 min. The expression of Bcl-2 and Bax was shown. Error bars represent the mean±S.E. (*n*=4). *P<0.01 *versus* *nt* siRNA+C (boiled rVvhA, 200pg/ml) or Cont (boiled rVvhA, 200pg/ml). ^#^*P*<0.01 *versus* *nt* siRNA+rVvhA or rVvhA. (**d**) INT-407 cells were incubated in the presence of rVvhA (50 pg/ml) for various times (0–120 min) and then isolated to cytosol and mitochondria fractions. COX IV and *β*-actin were used internal control for mitochodria and cytosolic fractions, respectively. Error bars represent the means±S.E. (*n*=5). *P<0.05 *versus* 0 min. (**e**) INT-407 cells transfected with siRNAs for non-targeting (*nt*) control and *NF-κBp65* were incubated with rVvhA (50 pg/ml) for 120 min. The expression of cytochrome *c* was shown. Error bars represent the mean±S.E. (*n*=4). *P<0.01 *versus nt* siRNA+C (boiled rVvhA, 200 pg/ml). ^#^*P*<0.01 *versus nt* siRNA+rVvhA. (**f**) INT-407 cells were incubated in the presence of rVvhA (50 pg/ml) for various times (0–120 min), and then total protein was extracted. The expression of Caspase-9 and Cleaved Caspase-3 were confirmed by western blotting with Caspase-9 and Cleaved Caspase-3 antibodies. Error bars represent the means±S.E. (*n*=4). *P<0.01 *versus* 0 min. INT-407 cells were transfected with siRNA for *NF-κBp65* (**g**) or pretreated with inhibitors of ERK (PD98059, 10 *μ*M) and JNK (SP600125, 10 *μ*M) (**h**) prior to rVvhA (50 pg/ml) exposure for 120 min. The expression of Caspase-9 and Cleaved Caspase-3 was shown. Error bars represent the mean±S.E. (*n*=4). *P<0.01 *versus* *nt* siRNA+C (boiled rVvhA, 200pg/ml) or Cont (boiled rVvhA, 200pg/ml). ^#^*P*<0.01 *versus* *nt* siRNA+rVvhA or rVvhA. INT-407 cells were incubated in the presence of rVvhA (50 pg/ml) for various times (0–120 min) and then harvested. Total protein was extracted and blotted with (**i**) CDK2 and CDK4 antibodies as well as (**j**) cyclin D1 and cyclin E antibodies. Error bars represent the means±S.E. (*n*=4). *P<0.05 *versus* 0 min. ROD, relative optical density

To further elucidate the rVvhA-induced apoptosis, INT-407 cells were exposed to 50 pg/ml of rVvhA for 3 h. rVvhA decreased Bcl-2 expression but increased Bcl-2-associated X protein (Bax) expression, suggesting that rVvhA treatment altered the balance of Bcl-2/Bax in a time-dependent manner (Figure 5a). In addition, rVvhA-induced decrease in Bcl-2 and increase in Bax were reversed by knockdown of *NF-κBp65* by siRNA (Figure 5b) or pretreatment with inhibitors for ERK (PD98059) and JNK (SP600125) (Figure 5c). Moreover, rVvhA induced cytochrome *c* release from mitochondria to cytosol (Figure 5d), which was inhibited by silencing of *NF-κBp65* by siRNA (Figure 5e), suggesting the involvement of NF-*κ*B at a key step of mitochondrial apoptosis during rVvhA treatment. Consistent with these results, rVvhA stimulated the expression of caspase-9 and cleaved caspase-3 cleavages (Figure 5f), which were blocked by knockdown of *NF-κBp65* by siRNA (Figure 5g) or pretreatment with the inhibitors of ERK (PD98059) or JNK (SP600125) (Figure 5h). Additionally, INT-407 cells were exposed to 50 pg/ml of rVvhA for 2 h to confirm the effects of rVvhA on the expression of cell cycle-related proteins. rVvhA treatment yielded significant decreases in the level of CDK2 and CDK4 expression (Figure 5i) as well as cyclin D1 and cyclin E expression (Figure 5j) in a time-dependent manner. We have further addressed whether p53 and Akt activation is involved in apoptotic signaling pathway induced by rVvhA. Knockdown of p53 by siRNA did not show any significant effect on apoptotic/necrotic cell death in rVvhA-treated INT-407 cells (Supplementary Figure S4A). In addition, rVvhA did not regulate phosphorylation of Akt as well as p53 expression (Supplementary Figure S4B), suggesting that rVvhA may have unique signaling pathway to regulate mitochondria-mediated apoptotic cell death.

## Discussion

In this study, we present new findings showing that rVvhA has the ability to induce cytotoxicity mainly via an apoptotic mechanism, through which rVvhA induces the aggregation of lipid raft molecules coupled with NOX2 to stimulate the ROS-dependent phosphorylation of PKC*α*/ERK/JNK, which is responsible for the activation of the NF-*κ*B pathway. First, we show that rVvhA is the relevant cytolysin in promoting the apoptosis pathway via lipid rafts. This result is in contrast to a previous report, which revealed that the effect of VvhA in promoting cytotoxicity is independent of the action of lipid rafts.^12^ Although the discrepancy with regard to the functional role of rVvhA may be due to differences in the concentration of the treated rVvhA, the cell types, and/or the experiment conditions, our data revealed that lipid rafts aggregation is clearly involved in rVvhA-induced apoptosis, whereas the functional role of lipid rafts in necrotic cells induced by rVvhA was relatively weak, suggesting that rVvhA acting through lipid rafts has a selective effect on apoptosis. In fact, it has been shown that the cytotoxic mechanism of rVvhA in endothelial, gastric, and hepatoma cells is closely related to its ability to induce apoptotic cell death.^24^ In addition to rVvhA, many studies have reported that several enteric bacterial pathogens, including *H. pylori* vacuolating toxins^25^ and the entero toxin *Clostridium perfringens*,^26^ may interact with a detergent-resistant cellular membrane (DRM) composed of relatively abundant cholesterol, using the lipid rafts as an initial attachment platform and therefore having a cytotoxic effect on intestinal physiological functions. Therefore, our results here suggest that lipid rafts are a functional mediator that initiates the virulence effect of rVvhA to induce apoptotic cell death.

Increasing evidence has suggested that lipid rafts are clustered to form a redox signaling platform through gp91^phox^ (NOX2) coupling with cytosolic factors that include p47 ^phox^ (NCF1), p67 ^phox^ (NCF1), and small GTPase Rac1^20, 21, 22^ and that these processes subsequently produce superoxides and other ROS.^20^ These ROS may be either direct or indirect mediators of intracellular signaling cascades that, among other actions, may induce the collapse of the mitochondrial membrane potential and trigger a series of mitochondria-associated events, including apoptosis. Although, in most cell types, mitochondrial ROS are thought to be the largest contributor to intracellular ROS production,^27^ our result showed that the sequestration cholesterol by M*β*CD attenuates intracellular ROS production and apoptosis induced by rVvhA. Hence, this finding further indicates that the epithelial ROS are generated by NADPH oxidase within lipid rafts and that this is initially associated with mitochondrial damage, resulting in the production of mitochondrial ROS and thereby contributing to increased total intracellular ROS generation for apoptosis.

We also showed that rVvhA induces an influx of Ca^2+^ on PKC activation, in that PKC is required for ROS production to induce apoptosis. Many pathogens have been shown to evoke the mobilization of Ca^2+^, leading to cytotoxic and myotoxic effects.^28^ Indeed, multifunctional autoprocessing RTX from *V. vulnificus* has the ability to regulate Ca^2+^ signaling during programed cell death.^29^ In addition, we and others have suggested that multiple signaling processes, such as those acting through the Ca^2+^ and PKC pathways, were rapidly activated in target cells through ROS^30, 31, 32^ and that these pathways are linked to bacterial stratagems to modulate the host signaling pathway.^33^ Interestingly, rVvhA uniquely activates conventional PKC*α* in PKC isoforms in INT-407 cells. Although novel PKC*ɛ* activation was found to require the apoptosis induced by bacterial lipopolysaccharide,^34^ many studies have established a critical role of conventional PKC*α* in apoptotic process during infection of EPEC or *C. perfringens*.^28, 35^ Particularly, PKC*α* activation in response to EPEC infection appears to be involved in impairing intestinal barrier function as well as causing apoptotic cell death in the host.^36, 37^ Hence, our results suggest that rVvhA has a pivotal role in Ca^2+^-dependent PKC*α* activation via ROS generation compounded by the recruitment of lipid rafts in intestinal epithelial cells. Interestingly, our results revealed that PKC acts to transduce ROS signals into ERK/JNK cascades. Despite the frequent involvement of p38 MAPK in the ROS signaling pathway induced by *H. pylori* infection,^4^ p38 MAPK did not respond to a treatment with rVvhA, implying a functional role of rVvhA in the determination of downstream targets. This indicates that *V. vulnificus* effectively causes intestinal apoptotic process by producing VvhA with modes of action that differ from *H. pylori*. On the other hand, we found that rVvhA can induce the phosphorylation of NF-*κ*B through ERK/JNK and that the inhibition of NF-*κ*B blocks rVvhA-induced apoptotic cell death. Regarding the role of MAPKs in NF-*κ*B activation, earlier work showed that the JNK pathway induced by ROS can influence NF-*κ*B activation in promoting apoptotic cell death.^38^ In addition, pERK1/2 was reported to have the ability to translocate into the nucleus, where it phosphorylates various substrates, such as transcriptional factors, thereby transmitting the signals received by cell surface receptors to the nucleus.^39^ Indeed, it has been previously shown that ERK regulates the activation of I*κ*B kinase and NF-*κ*B in macrophage stimulated by bacterial endotoxin, lipopolysaccharide.^40^ Hence, it is conceivable that ROS induced by rVvhA has a potential role in promoting the NF-*κ*B pathway through the activation of ERK and JNK. Based on these results, we suggest that rVvhA stimulates ROS-mediated PKC activation to activate NF-*κ*B-dependent signaling pathways via MAPKs in promoting INT-407 cell apoptosis.

Interestingly, rVvhA significantly induced a shift in the Bax/Bcl-2 ratio via the activation of NF-*κ*B. The ratio between Bcl-2 and Bax has been suggested as a primary determining factor of the degree of susceptibility to apoptosis.^41, 42, 43^ In addition, Bax is known to have a specific promoter region for binding the NF-*κ*B.^44, 45^ Thus it is possible that rVvhA may be related to the rectification of a disturbed Bcl-2/Bax balance by stimulating NF-*κ*B activation, which leads to the mitochondrial apoptotic pathway. On the other hand, exposure to cellular stress also can trigger another transcription factor p53 to induce apoptosis, where p53 pathway is negatively regulated by Akt activation.^46^ However, our results revealed that rVvhA-mediated apoptotic signal pathway was independent of p53 and Akt activation. Interestingly, the p53-independent apoptosis was known to regulate NF-*κ*B activation as well as Bcl-2 degradation, although mechanism is still enigmatic.^47^ Thus our results indicate that rVvhA controls Bcl-2/Bax balance by stimulating NF-*κ*B activation via p53-independent apoptotic pathway. We subsequently showed that the release of mitochondrial cytochrome *c* is a unique downstream event of the rVvhA-evoked mitochondrial apoptotic pathway accompanying the cleavage of caspase-9 and caspase-3. The translocation of Bax into the mitochondria is known to induce oligomer formation and mitochondrial membrane permeabilization, facilitating the release of mitochondrial cytochrome *c* as well as binding of caspase-activating proteins to pro-caspase-9 that are necessary for the processing and activation of downstream caspase activation.^48, 49^ Interestingly, other pore-forming alpha toxin from *Staphylococcus aureus* has been shown to induce massive necrosis without having apoptotic process,^50^ while EPEC was shown to disrupt the mitochondrial membrane potential, resulting in the release of cytochrome *c* and apoptosis.^5^ Thus these results imply that VvhA is a unique pore-forming toxin that has the ability to stimulate mitochondrial apoptotic pathway in intestinal epithelial cells. In support of rVvhA-mediated apoptotic signal pathways, our results also elucidate the potential role of rVvhA in the inhibition of the expression of cell-cycle-related proteins. It is not clear whether these additional effects of rVvhA in promoting apoptotic cell death are a sequential result of mitochondrial cell death or, alternatively, an independent process involving other cellular signaling events. However, it is clear that these signs of apoptosis are closely related to the cell cycle blockade. Thus our results indicate that rVvhA stimulates mitochondrial cell death by decreasing the expression of CDK2/4 and cyclin D1/E.

Collectively, our results suggest that rVvhA induces NF-*κ*B-dependent mitochondrial cell death via the production of lipid raft-dependent ROS. Thus highlighting the signaling pathways involved in the rVvhA-stimulated apoptosis pathway may provide potential targets for strategic modulations during *V. vulnificus* infections. In conclusion, rVvhA acting on lipid rafts induces NOX2-mediated ROS production, with this being necessary for PKC/ERK/JNK activation in intestinal epithelial cells. It thereby stimulates the NF-*κ*Bp65-mediated Bcl-2/Bax imbalance to facilitate the cytochrome-*c*-mediated caspase-9/-3 activation in promoting mitochondrial cell death.

## Materials and Methods

### Materials

Fetal bovine serum (FBS) was purchased from BioWhittaker Inc. (Walkersville, MO, USA). The following antibodies were purchased: p-PKC, caspase-9, cyclin D1, cyclin E, CDK2, CDK4, cleaved caspase-3, p53, and PKC antibodies (from Cell Signaling Technology, Danvers, MA, USA); NOX2 antibody (from BD Biosciences, Franklin Lakes, NJ, USA); NCF1 antibody (from LifeSpan Biosciences, Seattle, WA, USA); p-ERK1/2, ERK, p-JNK, JNK, p-p38, p38, p-NF-*κ*Bp65, NF-*κ*Bp65, p-AKT, AKT, *β*-actin, pan-cadherin (Pan-cad), PKC*α*, PKC*δ*, PKC*ζ*, Bcl-2, and Bax antibodies (from Santa Cruz Biotechnology, Paso Robles, CA, USA); and horseradish peroxidase (HRP)-conjugated goat anti-rabbit and goat anti-mouse immunoglobulin G (from Jackson Immunoresearch, West Grove, PA, USA). 2′, 7′-dichlorofluorescein diacetate (CM-H_2_DCFDA) was obtained from Invitrogen (Carlsbad, CA, USA). A23187, M*β*CD, NAC, PD98059, SP600125, and CTB were purchased from Sigma Chemical Company (St. Louis, MO, USA). All of the pharmacological inhibitors listed did not show any significant cytotoxic effects by themselves as confirmed by FACS analysis in each experiment. All other reagents were of the highest purity, commercially available, and were used as received.

### Cells

Human intestinal epithelial (INT-407) cells were kindly provided by Professor Sang Ho Choi (Seoul National University, Seoul, Korea) and were grown at 37 °C in 5% CO_2_ in *α*-Minimum Essential Medium supplemented with 10% FBS and antibiotics (10 units/ml penicillin G and 10 *μ*g/ml streptomycin). INT-407 cells have previously been used to evaluate the function of virulence factors of *V. vulnificus* in regulation of pro-inflammatory process,^51^ cytotoxic effect,^52^ and cell adherence ability.^53^

### siRNA transfection

Cells were grown until 75% of the surface of the plate and transfected for 36 h with either a siRNA specific for PKC*α*, NF-*κ*Bp65, p53 (GE Dharmacon, Lafayette, CO, USA) or non-targeting (nt) siRNA as a negative control (GE Dharmacon) with HiPerFect Transfection Reagent (Qiagen, Valencia, CA, USA) according to the manufacturer's instructions. The transient knockdown of *ERK1/2* and *JNK* was achieved by transfection with specific amounts of siRNA (100 nM) obtained from Cell Signaling. The siRNA efficacy for PKC*α*, ERK1/2, JNK, NF-*κ*Bp65, and p53 was determined by western blotting (Supplementary Figure S5).

### Purification of the recombinant protein (r) VvhA

To find the functional role of VvhA in INT-407 cells, we have prepared a recombinant protein of VvhA (rVvhA). The oligonucleotides were designed using the *V. vulnificus* MO6-24/O genomic sequence (GenBankTM accession number CP002469 and CP002470, www.ncbi.nlm.nih.gov).^54^ Briefly, the open reading frame of VvhBA was amplified by PCR using a pair of primers for VvhA (Supplementary Table S1) and cloned into a His6-tag expression vector, pET29a(+) (Novagen, Madison, WI, USA) to result in pKS1201 (Supplementary Table S2). *E. coli* BL21 (DE3) harboring the pKS1201 was grown in LB-ampicillin media at 37 °C until the cultures reached an A_600_ between 0.5 and 0.6. The temperature was lowered to 30 °C, and the protein expression was induced by treatment with 1 mM isopropyl-*β*-D-thiogalactopyranoside for 6 h. The cells were harvested by centrifugation at 5000 × *g* for 20 min at 4 °C. The cell pellets were resuspended buffer A (20 mM Tris-Cl, pH 8.0, and 500 mM NaCl), and the cell suspensions were ultrasonicated. The crude cell extracts were centrifuged at 16 000 × *g* for 30 min at 4 °C, and the supernatant was filtered using a 0.2-*μ*m Whatman Puradisc syringe filter (Whatman, International Ltd., Maidstone, Kent, UK) for isolating the soluble fraction. Cell lysate containing His6-tagged VvhBA protein was mixed with 1 ml of nickel-nitrilotriacetic acid agarose (Qiagen) for 1 h at 4 °C, and the mixture was loaded on Bio-Spin Chromatography Columns (Bio-Rad Laboratories, Hercules, CA, USA). The resin was washed with buffer A, and bound VvhBA protein was eluted with buffer A containing 300 mM imidazole. After purification, the homogeneity of VvhBA was assessed by 12% sodium dodecyl sulfate-polyacrylamide gel electrophoresis (SDS-PAGE) and Coomassie Blue staining. Purified proteins was dialyzed against 20 mM Tris-Cl, pH 8.0, concentrated to 0.3 mg/ml using Slide-A-Lyzer Dialysis Cassettes (Thermo Scientific, Hudson, NH, USA), and stored at −80 °C until use.

### MTT cell viability assay

Cell viability was determined using the conversion of 3-(4,5-dimethylthiazol-2-yl)-2,5-diphenyltetrazolium bromide (MTT) to formazan via mitochondrial oxidation. Cells were pretreated with the indicated inhibitors prior to rVvhA exposure for various times. MTT solution was then added to each well at a final concentration of 1 mg/ml per well, and the plates were incubated at 37 °C for another 2 h. After incubation, 150 *μ*l of dimethylsulfoxide (DMSO) was added to each well to dissolve the formazan formed, and the absorbance was read at 570 nm using a spectrophotometer.

### [^3^H]thymidine incorporation

The [^3^H]thymidine incorporation experiments were performed as previously described by Brett *et al.*^55^ Briefly, INT-407 cells were synchronized by serum starvation for 24 h and then exposed to 50 pg/ml rVvhA for 24 h. After the incubation period, 1 *μ*Ci of [methyl-^3^H]-thymidine (specific activity: 74 GBq/mmol, 2.0 Ci/mmol; Amersham Biosciences, Buckinghamshire, UK) was added to the cultures for 1 h at 37 °C. Cellular [^3^H]thymidine uptake was quantified by liquid scintillation counting of harvested cellular material (Wallac, Turku, Finland). All values were converted from absolute counts to percentages of control and reported as mean±S.E. of triplicate experiments.

### Flow cytometry

Cells were synchronized in the G_0_/G_1_ phase by culture in serum-free media for 24 h before incubation of rVvhA. The cell death of INT-407 cells was detected with an Annexin V and PI Staining Kit (BD Biosciences) according to the manufacturer's instructions. Briefly, the cells were detached with 0.05% trypsin/ethylenediaminetetraacetic acid (EDTA), and 1 × 10^5^ cells were resuspended with Annexin V-binding buffer (0.1 M 4-(2-hydroxyethyl)-1-piperazineethanesulfonic acid (HEPES)/NaOH (pH 7.4), 1.4 M NaCl, 25 mM CaCl_2_). And then the cells were stained with Annexin V (25 *μ*g/ml) and PI (125 ng/ml) and incubated for 15 min at room temperature in the dark. The sample was read by flow cytometry and analyzed using the CXP software (Beckman Coulter, Brea, CA, USA).

### Apoptosis/necrosis detection

INT-407 cells grown to confluence in 96-well plates were synchronized in the G_0_/G_1_ phase by culture in serum-free media for 24 h before incubation of rVvhA. The cell death of INT-407 cells was detected with an Apoptosis/Necrosis Detection Kit (Abcam, Cambridge, MA, USA) according to the manufacturer's instructions. Briefly, the cells were treated with Apopxin Green indicator as a phosphatidylserine marker and 7-AAD, a fluorescent membrane-impermeable red dye, and were then incubated in the dark for 60 min at room temperature. After the cells were rinsed with ice-cold phosphate-buffered saline (PBS), the level of cell death was examined using a luminometer (Victor3; Perkin-Elmer, Waltham, MA, USA) and quantified by measuring absorbance at excitation and emission wavelengths of 490 and 525 nm for detection of Apopxin Green Indicator or at excitation and emission wavelengths of 490 and 650 nm for detection of 7-AAD.

### Detergent-free purification of caveolin-rich membrane fraction

INT-407 cells grown to confluence in 100-mm dishes were used to prepare caveolin-enriched membrane fractions as described previously.^56^ Cells were washed twice with ice-cold PBS, scraped into 2 ml of 500 mM sodium carbonate (pH 11.0), transferred to a plastic tube, and homogenized with a Sonicator 250 apparatus (Branson Ultrasonic, Danbury, CT, USA) using three 20-s bursts. The homogenate was adjusted to 45% sucrose by the addition of 2 ml 90% sucrose prepared in 2-(*N*-morpholino) ethanesulfonic acid (MES)-buffered solution consisting of 25 mM MES-buffer solution (pH 6.5) and 0.15 M NaCl and placed at the bottom of an ultracentrifuge tube. A 5–35% discontinuous sucrose gradient was formed (4 ml each of 5% and 35% sucrose, both in MES-buffer solution containing 250 mM sodium carbonate) and centrifuged at 40 000 × *g* for 20 h in a Beckman SW41 Rotor (Beckman Coulter, Fullerton, CA, USA). Twelve fractions were collected and analyzed by 12% SDS-PAGE.

### Immunoprecipitation

Interaction of NOX with NCF1 or caveolin-1 was analyzed by immunoprecipitation and western blotting. Cells were lysed with lysis buffer (1% Triton X-100 in 50 mM Tris–HCl pH 7.4 containing 150 mM NaCl, 5 mM EDTA, 2 mM Na_3_VO_4_, 2.5 mm Na_4_PO_7_, 100 mm NaF, 200 nm microcystin lysine–arginine, and protease inhibitors). Cell lysates (400 *μ*g) were mixed with 10 *μ*g of each antibodies. The samples were incubated for 4 h, mixed with Protein A/G PLUS-agarose immunoprecipitation reagent (Pierce, Rockford, IL, USA) and then incubated for an additional 12 h. The beads were washed four times, and the bound proteins were released from the beads by boiling in SDS-PAGE sample buffer for 5 min. Samples were analyzed by western blotting.

### Western blotting analysis

Cells were harvested, washed twice with PBS, and lysed with buffer (20 mM Tris (pH 7.5), 1 mM EDTA, 1 mM ethylene glycol tetraacetic acid, 1% Triton X-100, 1 mg/ml aprotinin, and 1 mM phenylmethylsulfonylfluoride) for 30 min on ice. The lysates were then cleared by centrifugation (22 250 × *g* at 4 °C for 30 min). Protein concentration was determined by the Bradford method.^57^ Equal amounts of protein (20 *μ*g) were resolved by 10% SDS-PAGE and transferred to a polyvinylidene fluoride membranes. The membranes were washed with Tris-buffer solution-Tween 20 solution (10 mM Tris-HCl (pH 7.6), 150 mM NaCl, and 0.05% Tween-20), blocked with 5% skim milk for 1 h, and incubated with appropriate primary antibody at 4 °C for overnight. The membrane was then washed and detected with a horseradish peroxidase-conjugated secondary antibody. The bands were visualized by enhanced chemiluminescence (Amersham Pharmacia Biotech Inc., Buckinghamshire, UK). The relative optical density of the bands was quantified using the Scion Imaging Software (Scion Image Beta 4.02, Frederick, MD, USA).

### Confocal microscopy

INT-407 cells were fixed in 4% paraformaldehyde in PBS for 10 min at room temperature, permeabilized in 0.2% Triton X-100 in PBS for 5 min, and blocked in PBS containing 5% normal goat serum for 30 min at room temperature. Cells were then stained with primary antibody for overnight at 4 °C. Following three washes with PBS, the cells were incubated with antibodies for caveolin-1, PKC*α*, and NF-*κ*B or Alexa 488-conjugated CTB, counterstained with PI in PBS containing 1% (v/v) BSA, and washed three times for 10 min each with PBS. Samples were mounted on slides and visualized with an Olympus FluoView 300 confocal microscope (Olympus, Tokyo, Japan) with × 400 objective.

### Measurement of intracellular ROS production

CM-H_2_DCFDA (DCF-DA), which acts as a ROS-sensitive fluorophore, was used to detect the general ROS production. DCF-DA (10 *μ*M) was added to cells, which were then incubated in the dark for 30 min at room temperature. Cells were then viewed using the FluoView 300 confocal microscope (Olympus) with a × 200 objective for imaging, the fluorescence was excited at 488 nm, and the light emitted was observed at 515–540 nm. In order to quantify the intracellular ROS levels, the cells treated with DCF-DA were rinsed twice with ice-cold PBS and then scraped. A 100-*μ*l cell suspension was loaded into a 96-well plate and examined using a luminometer (Victor3; Perkin-Elmer) and a fluorescent plate reader at excitation and emission wavelengths of 485 and 535 nm, respectively.

### Subcellular fractionation

Harvested cell pellets were mixed with buffer 1 (250 mM sucrose, 50 mM Tris-HCl, 5 mM MgCl_2_) in the presence of protease inhibitor cocktail (Pierce) and incubated for 10 min on an end-over-end shaker and centrifuged at 1000 × *g* for 10 min. The supernatants with cytosolic protein were transferred to iced tubes. The pellet was suspended in buffer 2 (1 M sucrose, 50 mM Tris-HCl, 5 mM MgCl_2_) for 30 min and centrifuged at 6000 × *g* for 10 min, and the supernatants containing membrane proteins were transferred to new tubes. The remaining pellet was suspended in buffer 3 (20 mM Tris-HCl, 0.4 M NaCl, 15% glycerol, 1.5% Triton X-100) with protease inhibitor cocktail and incubated for 10 min on an end-over-end shaker. After centrifugation at 6800 × *g* for 10 min, the supernatants was collected and designed as the nuclear proteins.

### Measurement of calcium influx

Changes in intracellular calcium concentrations were monitored using Fluo-3-AM that had initially been dissolved in DMSO. Cells in 35-mm diameter culture dishes were rinsed with a bath solution (140 mM NaCl, 5 mM KCl, 1 mM CaCl_2_, 0.5 mM MgCl_2_, 10 mM glucose, 5.5 mM HEPES (pH 7.4)) and were then incubated in a bath solution containing 2 *μ*M Fluo-3-AM for 40 min, rinsed, mounted on a perfusion chamber, and scanned at 1-s intervals using Olympus FluoView 300 confocal microscope with × 300 objective. The fluorescence was produced by excitation at 488 nm, and the emitted light was observed at 515 nm. All analyses of calcium influx were processed in a single cell, and the results are expressed as the fluorescent intensity (*F*/*F*0%, arbitrary unit, where *F* is fluorescence captured at a particular time and *F*0 is the initial fluorescence image captured).

### Cytosol and mitochondria fractionation

Isolation of mitochondria from cultured INT-407 cells was performed by using the Mitochondria Isolation Kit (Thermo Fisher Scientific Inc., Rockford, IL, USA) according to the manufacturer's instructions. Briefly, INT-407 cells were harvested, and 800 *μ*l of mitochondria isolation reagent A containing the protease inhibitor was added. After addition of 10 *μ*l of reagent B and 800 *μ*l reagent C containing the protease inhibitor, they were centrifuged at 700 × *g* for 10 min at 4 °C. The supernatant were centrifuged at 12 000 × *g* for 15 min at 4 °C. The supernatant contains cytosol fraction and the pellet contains the isolated mitochondria. For isolated mitochondria lysis, 100 *μ*l of 2% CHAPS in Tris-buffer solution (25 mM Tris, 0.15 M NaCl (pH 7.2)) was added to the mitochondrial pellet. And then they were centrifuged at 12 000 × *g* for 2 min. The fractions were subjected to western blotting.

### Statistical analysis

Results are expressed as means±S.Es. All experiments were analyzed by ANOVA, followed, in some cases, by a comparison of treatment means with the control using the Bonferroni–Dunn test. Differences were considered statistically significant at *P*<0.05.

## Abbreviations

CTB: cholera toxin B subunit
EDTA: ethylenediaminetetraacetic acid
ERK: extracellular signal-regulated kinase
FBS: fetal bovine serum
HEPES: 4-(2-hydroxyethyl)-1-piperazineethanesulfonic acid
JNK: c-Jun N-terminal kinase
MAPK: mitogen-activated protein kinase
M*β*CD: methyl-*β*-cyclodextrin
NAC: *N*-acetylcysteine
NF-*κ*B: nuclear factor-kappa B
Pan-cad: pan-cadherin
PBS: phosphate-buffered saline
PI: propidium iodide
PKC: protein kinase C
ROS: reactive oxygen species
SDS-PAGE: sodium dodecyl sulfate-polyacrylamide gel electrophoresis
Veh: vehicle
