# Supplementary material for: Vibrio vulnificus VvhA induces NF-κB-dependent mitochondrial cell death via lipid raft-mediated ROS production in intestinal epithelial cells
Source: Cell Death Dis. 2015 Feb 19;6(2):1655–. doi: 10.1038/cddis.2015.19 (PMC4669806; doi:10.1038/cddis.2015.19)
Supplement: Supplementary Table S1 [file cddis201519x1.doc]

**Supplemental Table 1** . Oligonucleotides used in this study

| **Name** | **Oligonucleotide Sequence (5' → 3')^a, b^** | **Use** |
| --- | --- | --- |
| **For GbpA overexpression** | | |
| Vvh-F | GGGCATATGCTTAATAACAAAAATAGAAATGTAG | Amplification of *VvhBA* ORF |
| Vvh-R | AAAACTCGAGTTTGACTTGTTGTAATGTGG |  |

^a^ The oligonucleotides were designed using the *V. vulnificus* MO6-24/O genomic sequence (GenBank^TM^ accession number CP002469 and CP002470, www.ncbi.nlm.nih.gov).

^b^ Regions of oligonucleotides not complementary to the corresponding genes are underlined
