# Supplementary material for: Vibrio vulnificus VvhA induces NF-κB-dependent mitochondrial cell death via lipid raft-mediated ROS production in intestinal epithelial cells
Source: Cell Death Dis. 2015 Feb 19;6(2):1655–. doi: 10.1038/cddis.2015.19 (PMC4669806; doi:10.1038/cddis.2015.19)
Supplement: Supplementary Table S2 [file cddis201519x2.doc]

**Supplemental Table 2**. Plasmids and bacterial strains used in this study

| **Strain or plasmid** | **Relevant characteristics ^a^** | **Reference or source** |
| --- | --- | --- |
| **Bacterial strains** | |  |
| *E. coli* |  |  |
| BL21 (DE3) | F^-^ *ompT* *hsdS_B_* (r_B_^-^m_B_^-^) *gal dcm* (DE3) | Laboratory collection |
| **Plasmids** |  |  |
| pET29a(+) | His_6_ tag fusion expression vector; Km^r^ | Novagen |
| pKS1201 | pET29a(+) with VvhBA; Km^r^ | This study |

^a^ Km^r^, kanamycin resistan
