# Supplementary material for: Vibrio vulnificus VvhA induces NF-κB-dependent mitochondrial cell death via lipid raft-mediated ROS production in intestinal epithelial cells
Source: Cell Death Dis. 2015 Feb 19;6(2):1655–. doi: 10.1038/cddis.2015.19 (PMC4669806; doi:10.1038/cddis.2015.19)
Supplement: Supplementary Figure S1 [file cddis201519x3.doc]

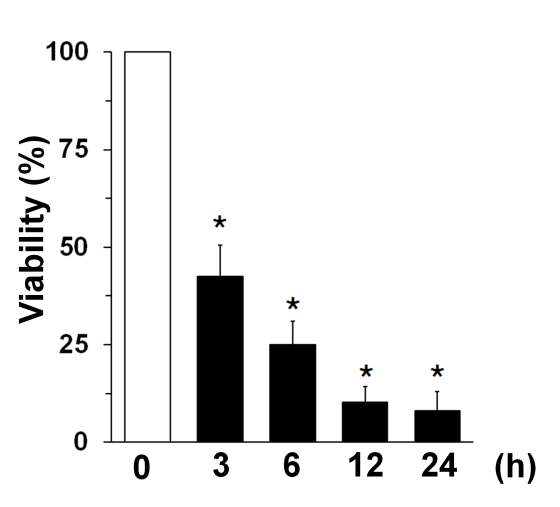


**Supplemental Figure 1**. **rVvhA induces cytotoxicity.** Time responses of 50 pg/mL of rVvhA in MTT assay are shown. Error bars represent the means ± S.E. n = 5. *, P < 0.01 versus 0 h.
