# Supplementary material for: Vibrio vulnificus VvhA induces NF-κB-dependent mitochondrial cell death via lipid raft-mediated ROS production in intestinal epithelial cells
Source: Cell Death Dis. 2015 Feb 19;6(2):1655–. doi: 10.1038/cddis.2015.19 (PMC4669806; doi:10.1038/cddis.2015.19)
Supplement: Supplementary Figure S2 [file cddis201519x4.doc]

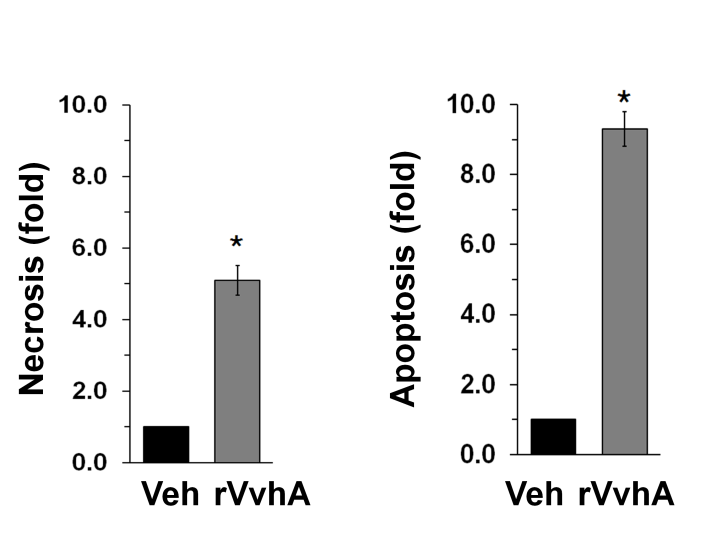


**Supplemental Figure 2**. **rVvhA induces necrotic cell death as well as apoptosis.** INT-407 cells were incubated with 50 pg/mL of rVvhA for 120 min. Quantitative analysis of the fold changes of apoptotic and necrotic cells by Apoptosis/ Necrosis Detection kit is shown. Error bars represent the means ± S.E. (*n* = 5). *, P < 0.05 versus Veh (boiled rVvhA, 200 pg/mL).
