# Supplementary material for: Vibrio vulnificus VvhA induces NF-κB-dependent mitochondrial cell death via lipid raft-mediated ROS production in intestinal epithelial cells
Source: Cell Death Dis. 2015 Feb 19;6(2):1655–. doi: 10.1038/cddis.2015.19 (PMC4669806; doi:10.1038/cddis.2015.19)
Supplement: Supplementary Figure S3 [file cddis201519x5.doc]

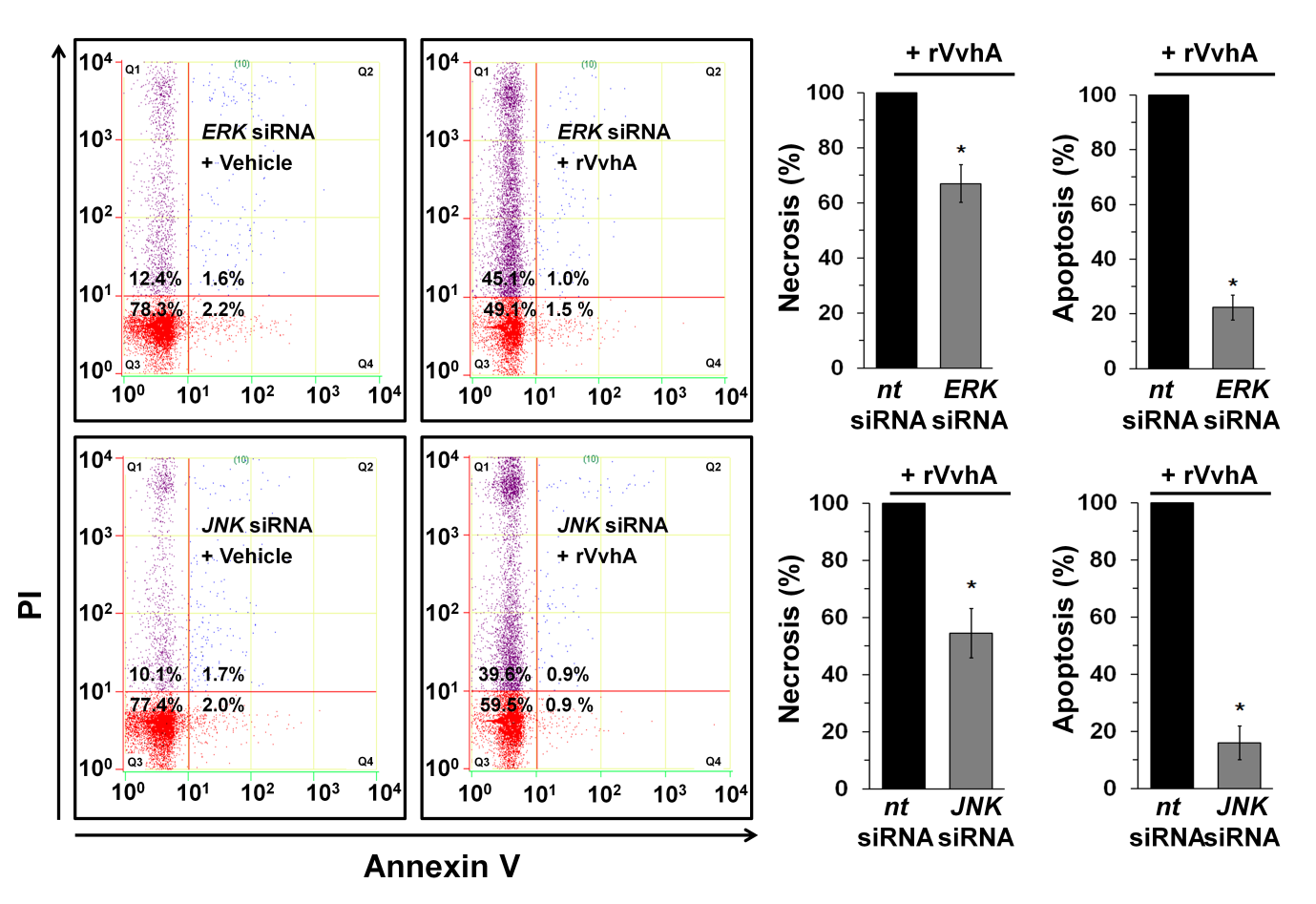


**Supplemental Figure 3**. **Involvement of ERK and JNK in rVvhA-induced cell death.** INT-407 cells transfected with siRNAs for *ERK1/2* and *JNK* were incubated with rVvhA (50 pg/mL) for 120 min. Percentages of necrosis, survival, and apoptosis were measured by using PI/Annexin V staining and flow cytometry (left panels). Quantitative analysis of the percentage of necrotic (Q1) and apoptotic (Q2+Q4) cells by FACS analysis is shown (right panels). Error bars represent the means ± S.E. (n = 4). *, P < 0.01 versus *nt* siRNA + rVvhA.
