# Supplementary material for: Vibrio vulnificus VvhA induces NF-κB-dependent mitochondrial cell death via lipid raft-mediated ROS production in intestinal epithelial cells
Source: Cell Death Dis. 2015 Feb 19;6(2):1655–. doi: 10.1038/cddis.2015.19 (PMC4669806; doi:10.1038/cddis.2015.19)
Supplement: Supplementary Figure S4 [file cddis201519x6.doc]

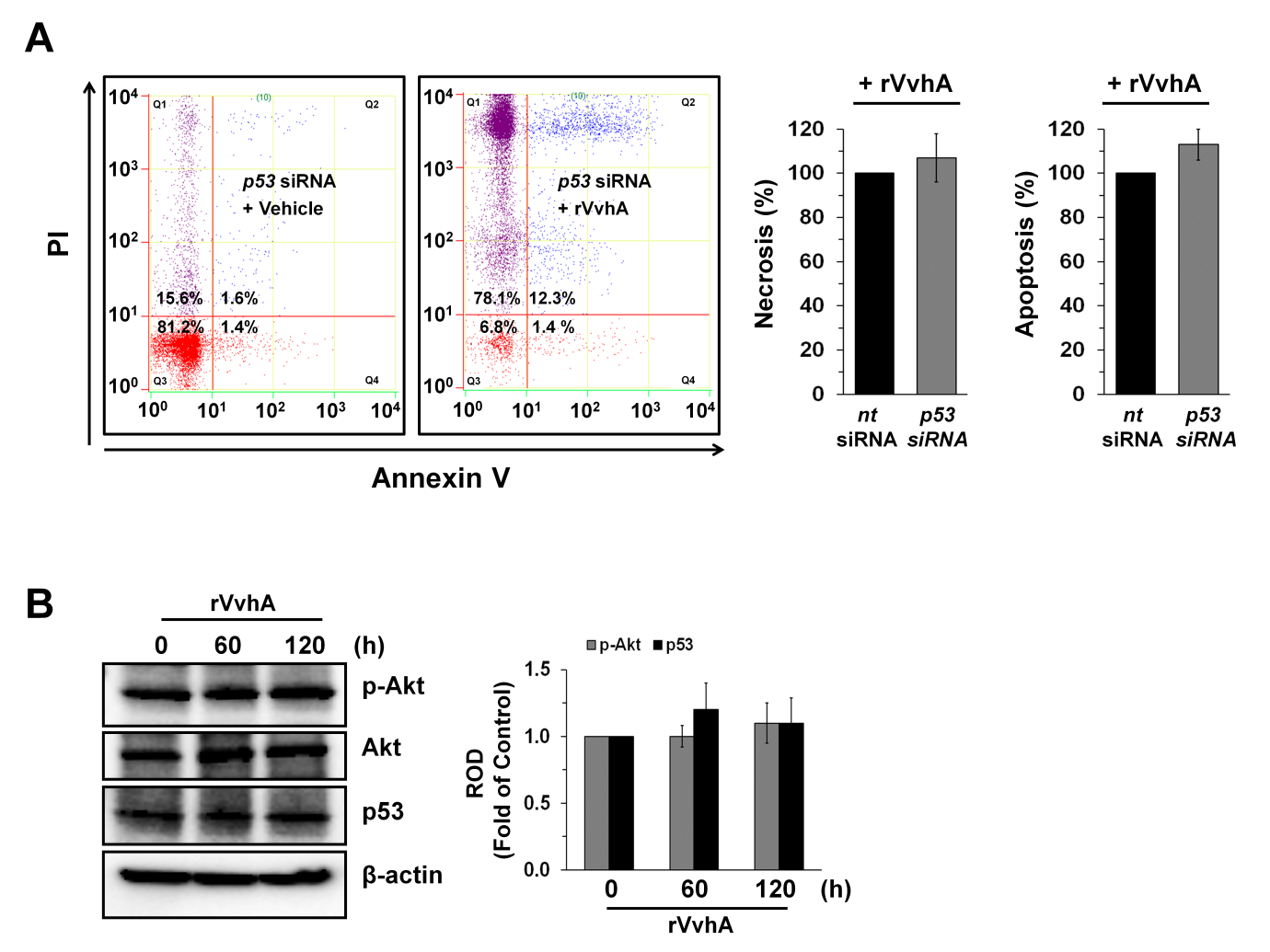


**Supplemental Figure 4**. **rVvhA did not regulate activation of Akt as well as p53.** (A) INT-407 cells transfected with siRNAs for *p53* were incubated with rVvhA (50 pg/mL) for 120 min. Percentages of necrosis, survival, and apoptosis were measured by using PI/Annexin V staining and flow cytometry (left panels). Quantitative analysis of the percentage of necrotic (Q1) and apoptotic (Q2+Q4) cells by FACS analysis is shown (right panels). Error bars represent the means ± S.E. (*n* = 4). (B) Phosphorylation of Akt and expression of Akt and p53 in cells treated with rVvhA are shown. *n* = 3.
