# Supplementary material for: Vibrio vulnificus VvhA induces NF-κB-dependent mitochondrial cell death via lipid raft-mediated ROS production in intestinal epithelial cells
Source: Cell Death Dis. 2015 Feb 19;6(2):1655–. doi: 10.1038/cddis.2015.19 (PMC4669806; doi:10.1038/cddis.2015.19)
Supplement: Supplementary Figure S5 [file cddis201519x7.doc]

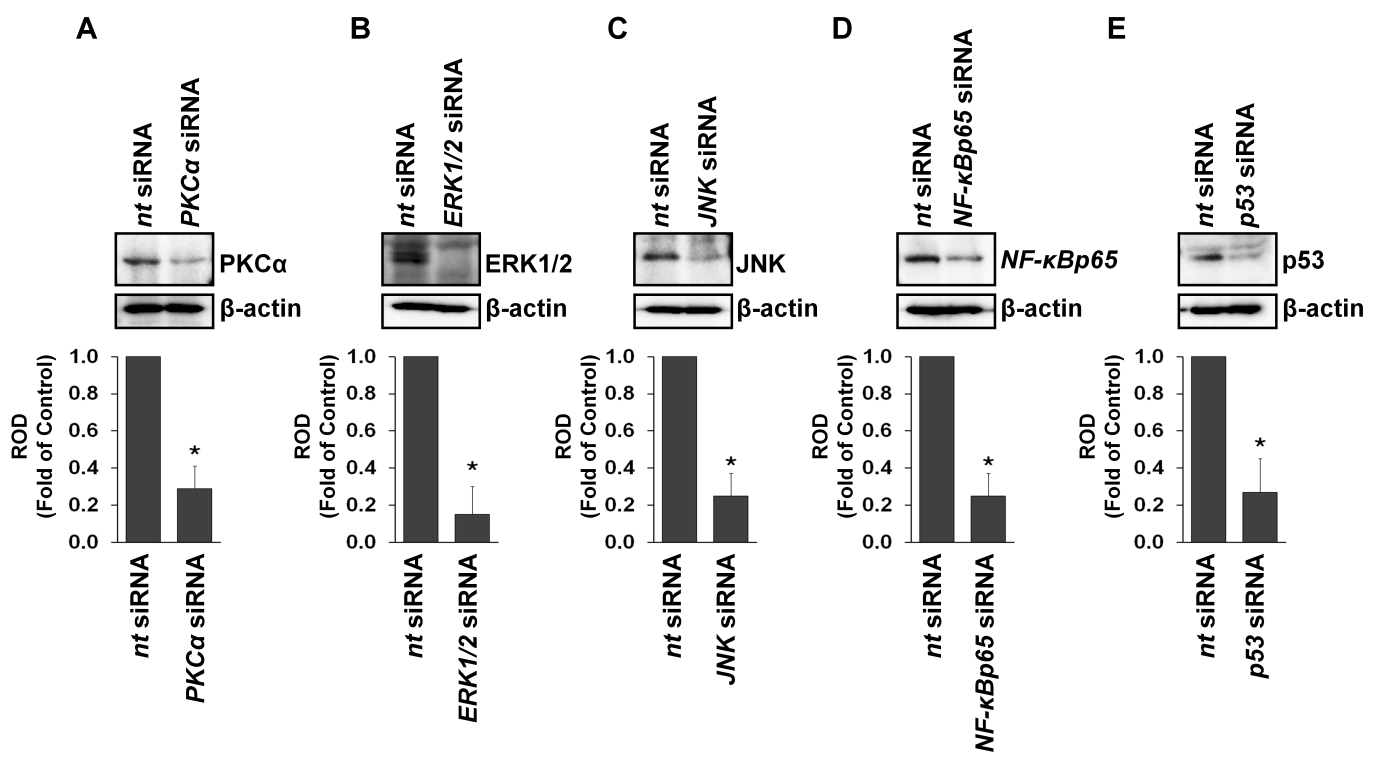


**Supplemental Figure 5. Effect of siRNA on target proteins.** Cells were transfected for 36 h with *PKCα, ERK1/2, JNK, NF-κBp65, and p53* or *non-targeting* (*nt*) siRNA using HiPerFect Transfection Reagent. Protein expressions were analyzed by using Western blot. The knockdown efficacies of PKCα (A), ERK1/2 (B), JNK (C), NF-κBp65 (D), and p53 (E) were 71%. 85%, 75%, 69%, and 73%. Error bars represent the means ± S.E. from three independent experiments. *, *P <* 0.05 vs *nt* siRNA
